# Supplementary material for: 3D single-molecule super-resolution microscopy with a tilted light sheet
Source: Nat Commun. 2018 Jan 9;9:123. doi: 10.1038/s41467-017-02563-4 (PMC5760554; doi:10.1038/s41467-017-02563-4)
Supplement: Supplementary file 1 — Supplementary Information [file 41467_2017_2563_MOESM1_ESM.pdf]

Supplementary Figures

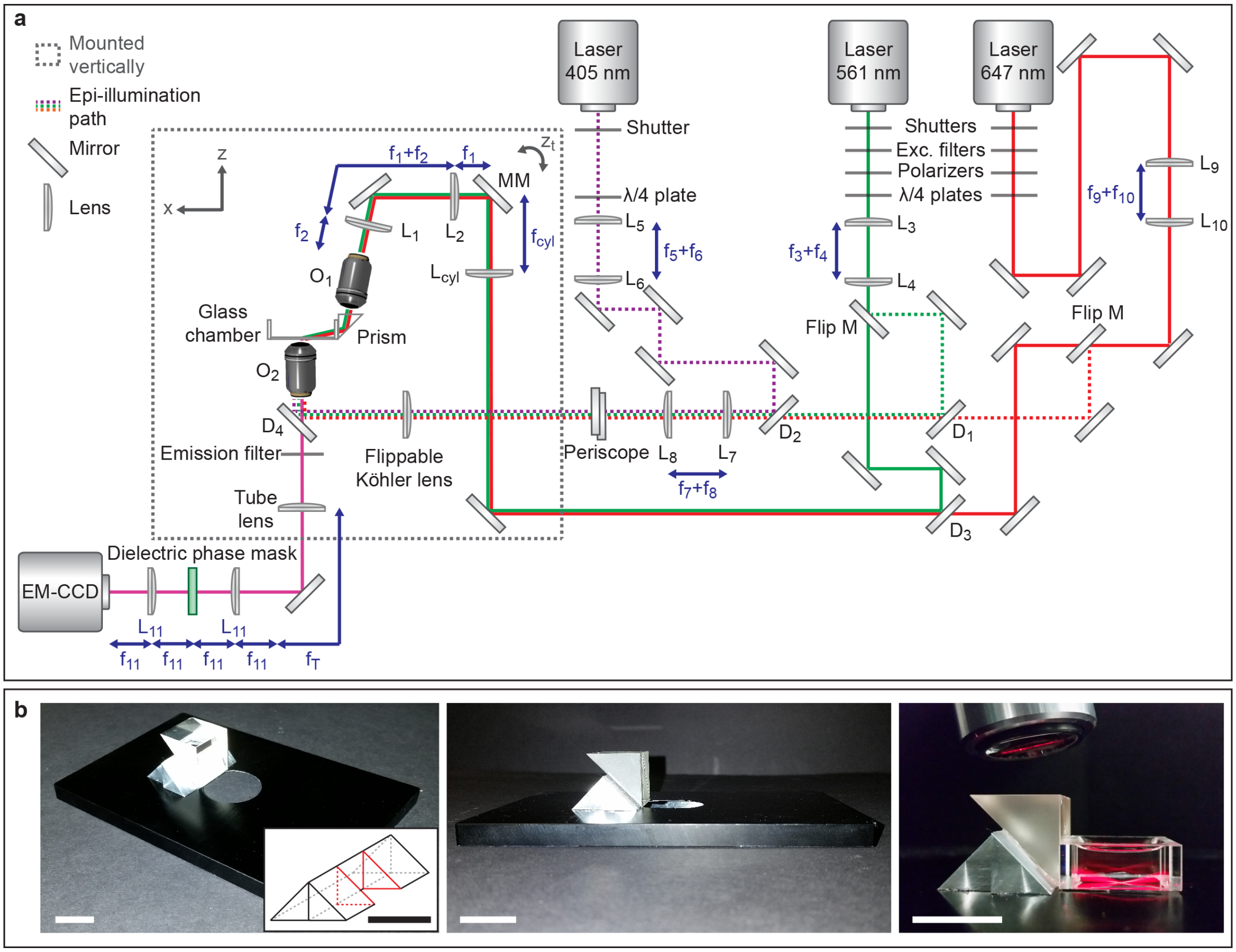

Supplementary Figure 1 Illustrations of the imaging platform. **a** Detailed schematic of the optical setup shown for a single-channel 4*f* setup with a transmissive dielectric phase mask. Tilting the motorized mirror (MM) in the direction given by the gray, semicircular arrow, z_t, indicates translation of the light sheet in z. Translation in y is performed by tilting the mirror in the perpendicular direction. L_1-L_{11}: lenses, f_1-f_{11}: focal lengths of lenses, Flip M: flip mirror, D_1-D_4: dichroic mirrors, L_{cyl}: cylindrical lens, f_{cyl}: focal length of cylindrical lens, O_1: illumination objective, O_2: detection objective. **b** Photos of the glass prism attachment on the microscope stage. The glass prism is attached to a right triangular aluminum prism with a hole cut out of the back to create an air-glass interface for total internal reflection to occur (inset schematic shows the Al support). The rightmost photo shows the light sheet being reflected by the prism into a fluorescent solution (1:1000 Alexa Fluor 647 conjugated secondary antibodies (Abcam, ab150067) in water) in the sample chamber. Scale bars are 2 cm.

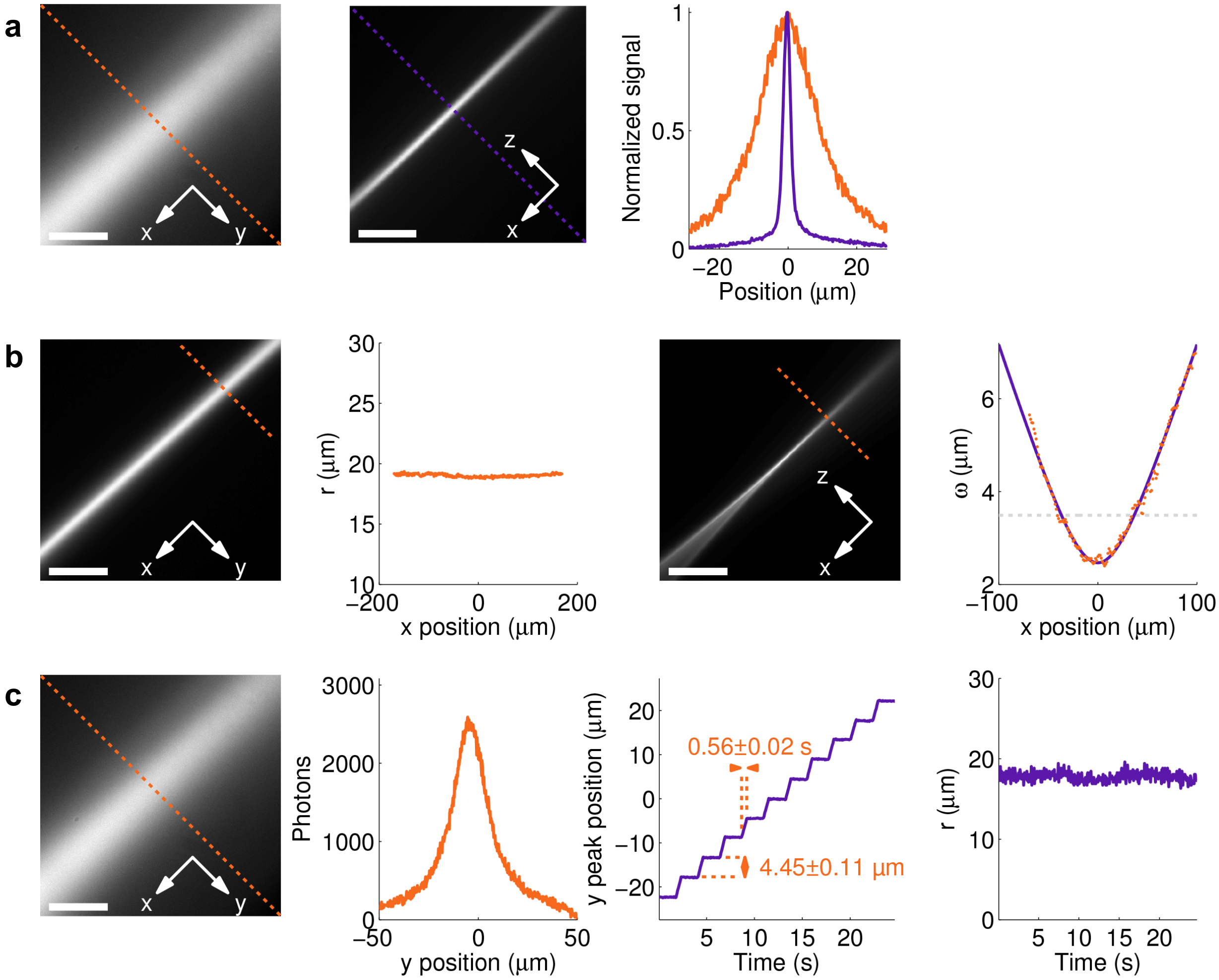

Supplementary Figure 2 Light sheet characteristics**. a** Images showing the width, r, and thickness, ω, of the light sheet. The graph shows line scans along y and z for estimation of width and thickness, respectively, using the axis definitions in **Figure 1**. The thickness and width were estimated from 1D Gaussian fits to the line scans. **b** To estimate the width variation (left) and the confocal parameter (right) along the direction of beam propagation, x, the light sheet was imaged using a 20x objective. The light sheet profiles were determined by measuring the cross-sectional intensity along different parts of the light sheet in y and z (orange dots in graphs on right). The widths were estimated from 1D Gaussian fits to the line scans. The thickness dependence was well described using the Gaussian beam propagation equation (purple line), from which the confocal parameter was estimated. **c** To estimate the relationship between the step size of the motorized mirror and the translation of the light sheet along y in the sample plane the mirror was scanned in 1000 steps intervals (left). The cross-sectional profile of the light sheet was measured by intensity line scanning of the resulting images in y (mid left), and 1D Gaussian fitting of the line scans was used to estimate the center position of the light sheet as a function of time throughout the scanning of the motorized mirror (mid right). The width of the light sheet was estimated throughout the scan over a total translation of 45 μm (right). In all measurements the light sheet profiles were imaged in a fluorescent solution (1:1000 Alexa Fluor 647 conjugated secondary antibodies (Abcam, ab150067) in water). The thickness (z) was imaged by rotating the cylindrical lens by 90°. Scale bars are 20 μm in **a** and **c**, and 100 μm in **b**.

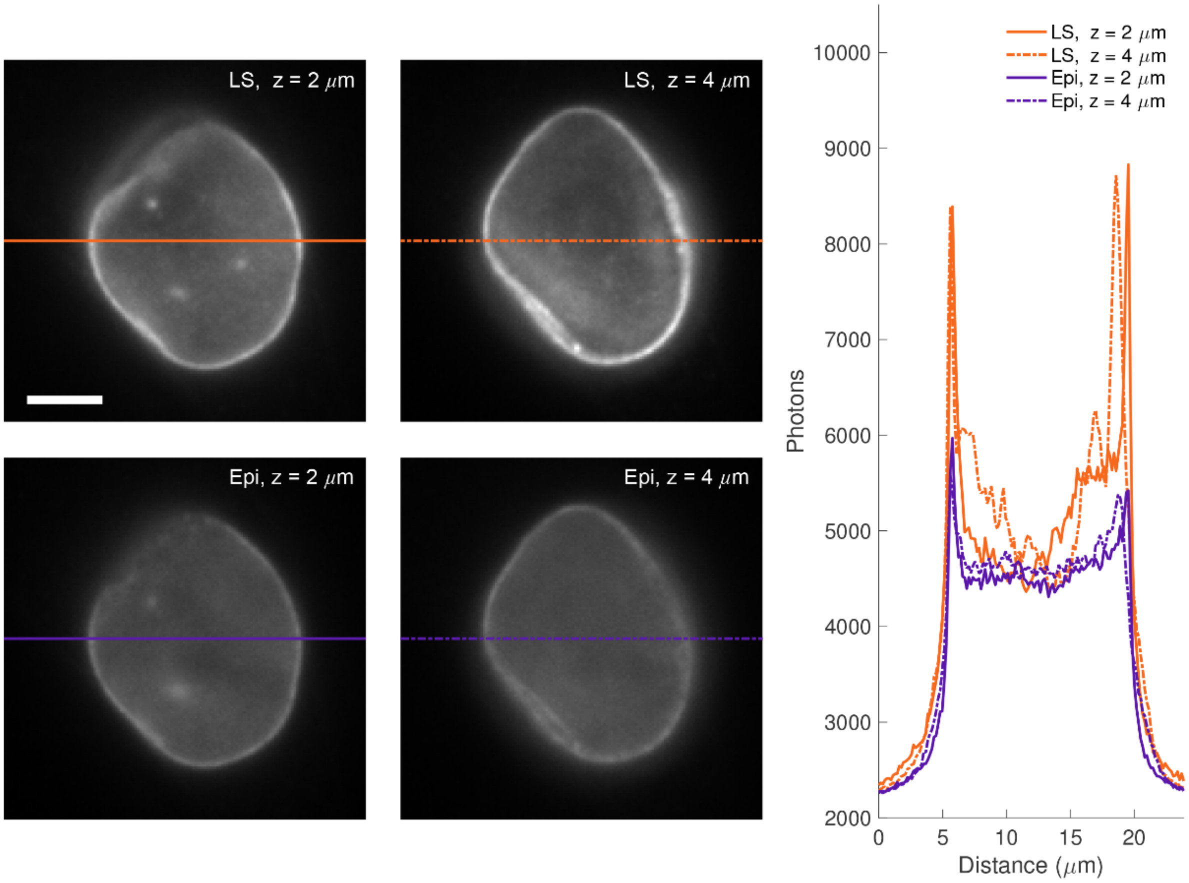

Supplementary Figure 3 Comparing different cell slices by axial translation of light sheet. The light sheet was moved together with the image plane using a motorized mirror, which allowed for sectioning throughout the entire thickness of a mammalian cell. Images show comparisons between light sheet (LS) and epi- (Epi) illumination at 2 μm and 4 μm from the coverslip. The graph shows line scans over the corresponding lines in the images, showing that the peak brightness and contrast are the same for different slices. All images show lamin B1 immunolabeled with Alexa Fluor 647 in a HeLa cell. All images are shown with the same linear grayscale. Scale bar is 5 μm.

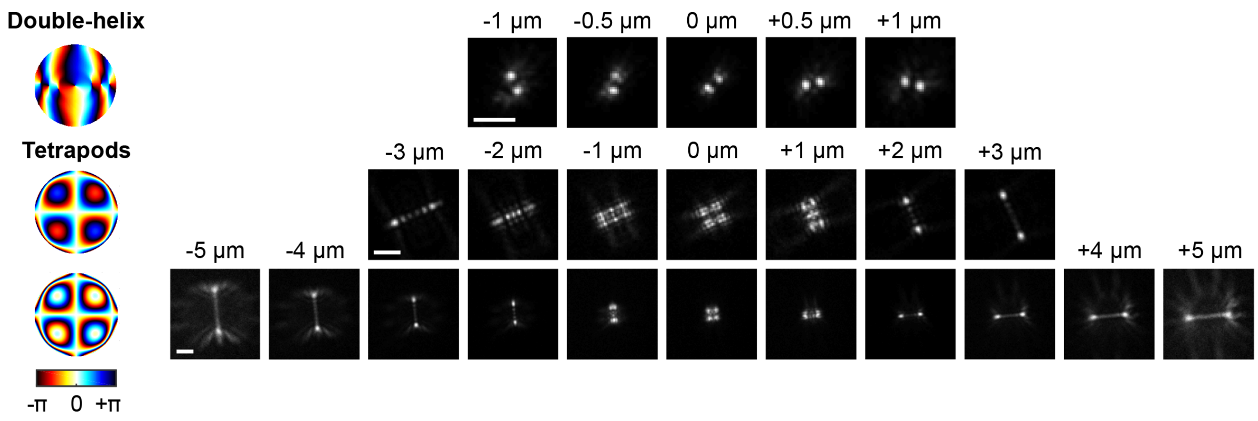

Supplementary Figure 4 Phase patterns and experimental point spread functions (PSFs)**.** Theoretical phase patterns used to generate the double-helix (DH) PSF, which has an axial range of ~2-3 µm, and Tetrapod PSFs with axial ranges of 6 µm (middle) and 10 µm (bottom). The DH and the 6-µm Tetrapod phase patterns were implemented using a transmissive dielectric phase mask, while the pattern for the 10-µm Tetrapod PSF was implemented using a deformable mirror. PSF images show a fluorescent bead on a cover slip imaged using the different phase masks while scanning the nominal focus to the positions indicated above the images. Scale bars are 3 µm.

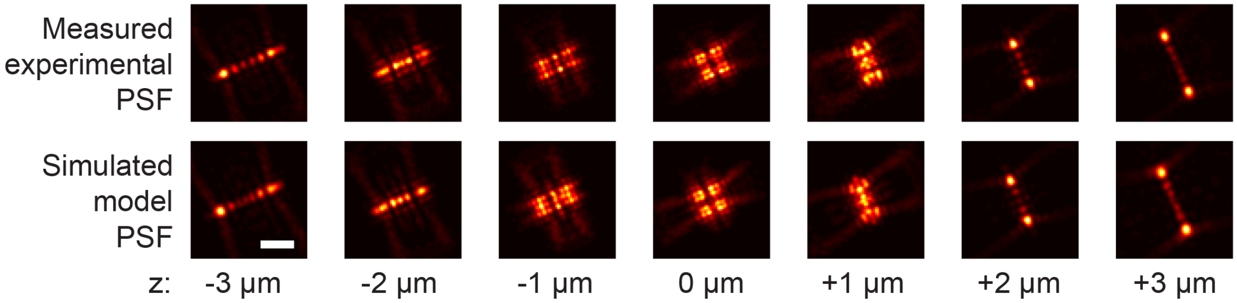

Supplementary Figure 5 Experimental and model point spread functions (PSFs)**.** Images showing the experimentally measured (top) and computationally modeled (bottom) PSF of a single emitter using the 6-µm Tetrapod phase pattern shown in **Figure 1**. Scale bar is 3 µm.

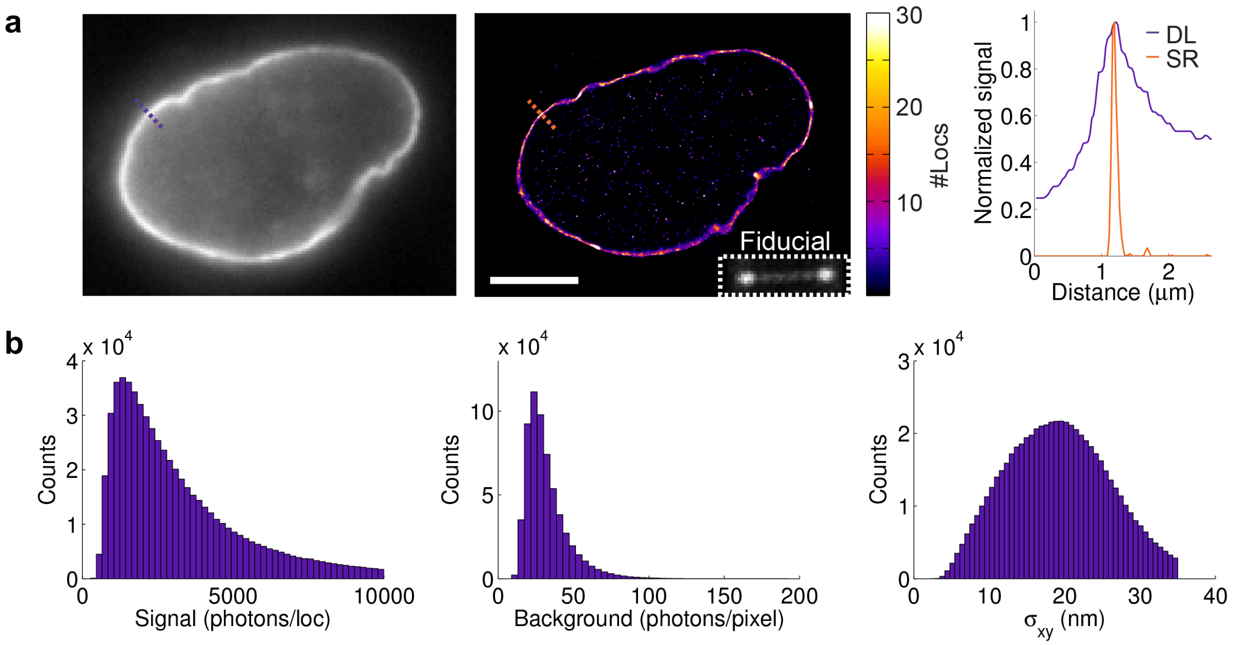

Supplementary Figure 6 2D imaging of nuclear lamina**. a** Diffraction-limited (DL) image and 2D super-resolution (SR) reconstruction of the nuclear lamina (lamin B1) in a HeLa cell. Inset shows the fiducial bead imaged with a 10-µm Tetrapod PSF used for real-time axial drift correction. The graph shows line scans at the corresponding dotted lines in the figures. By fitting the SR line scan with a 1D Gaussian function, the thickness of the nuclear lamina (FWHM) was determined to be 130 nm in the SR reconstruction. Scale bar is 5 µm. **b** Histograms showing the signal photons per localization, background photons per pixel, and xy localization precision of the data shown in **a** acquired using the standard point spread function. The data was filtered to remove localizations with a number of photons per localization higher than 10,000 and localization precision larger than 35 nm. This resulted in ~600,000 filtered localizations with median photons per localization of 2,530, background photons per pixel of 28, and localization precision of 19 nm.

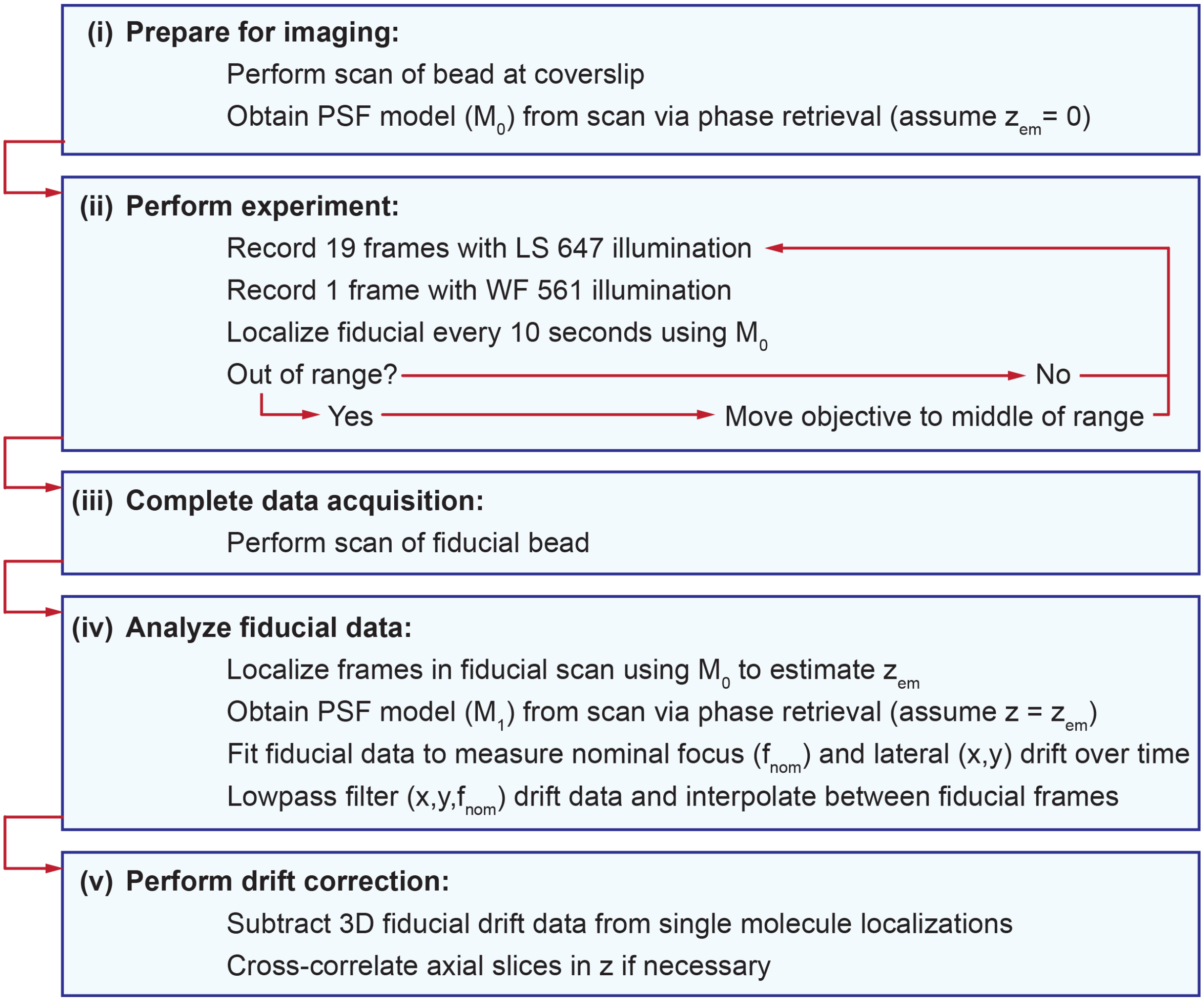

Supplementary Figure 7 Tetrapod analysis methodology**.** Flow chart showing the analysis methodology used for fiducial bead localization using the Tetrapod point spread functions (PSFs).

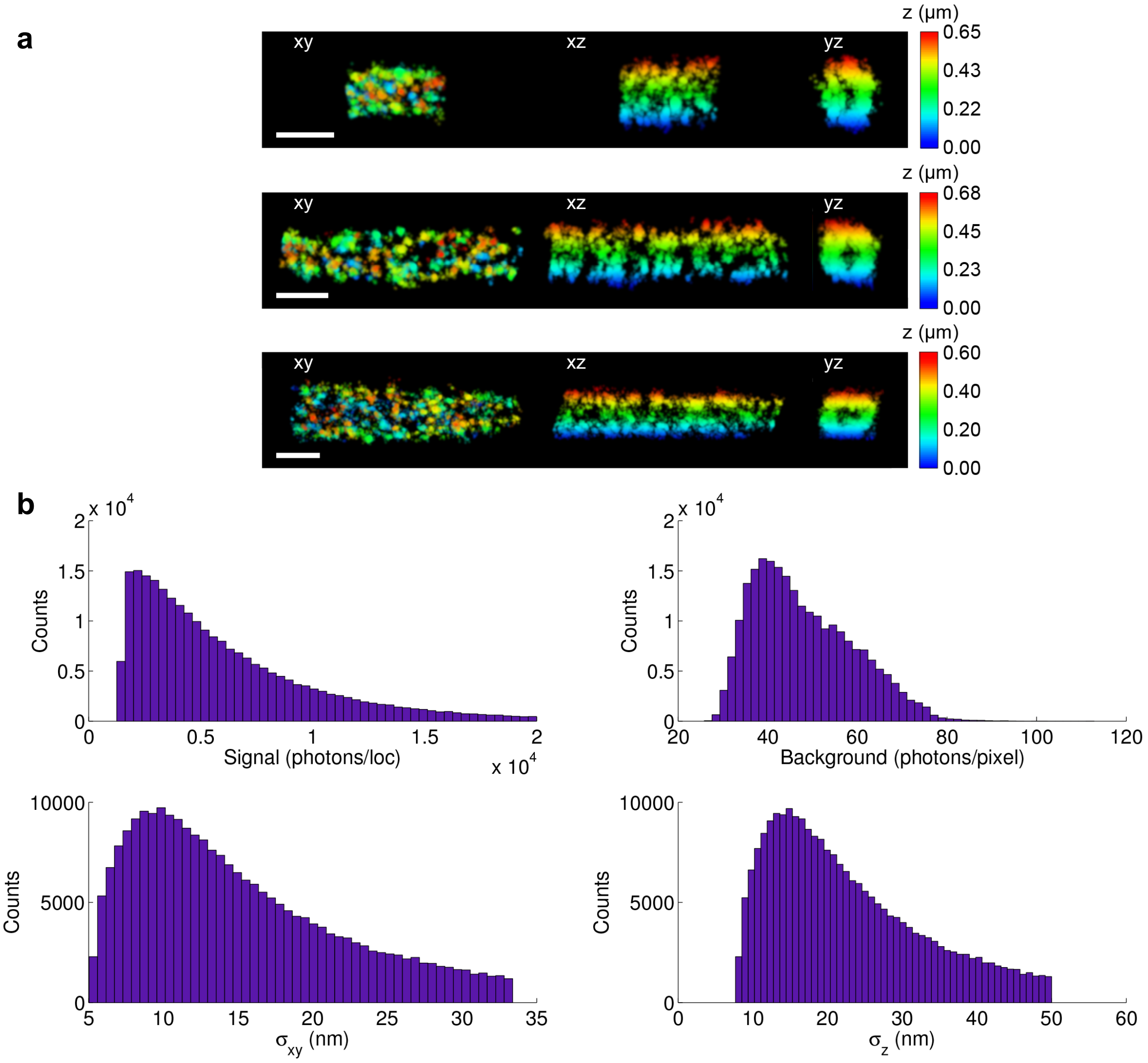

Supplementary Figure 8 Imaging of mitochondrial outer membrane using the double-helix PSF**. a** 3D super-resolution reconstructions of three mitochondria (TOM20) in HeLa cells immunolabeled with Alexa Fluor 647, shown in xy, xz, and yz views. Imaging of single molecules and fiducial fluorescent beads was performed using the double-helix PSF. Scale bars are 500 nm. **b** Histograms showing the signal photons per localization, background photons per pixel, and xy and z localization precision from the data shown in **a** and in **Figure 4a, b**. The data was filtered to remove localizations with a number of photons per localization higher than 20,000, distance between lobes smaller than 6 pixels and larger than 7.5 pixels, and z localization precision larger than 50 nm. This resulted in ~240,000 filtered localizations with median photons per localization of 4,857, background photons per pixel of 46, and localization precision of 13 nm and 20 nm in xy and z, respectively.

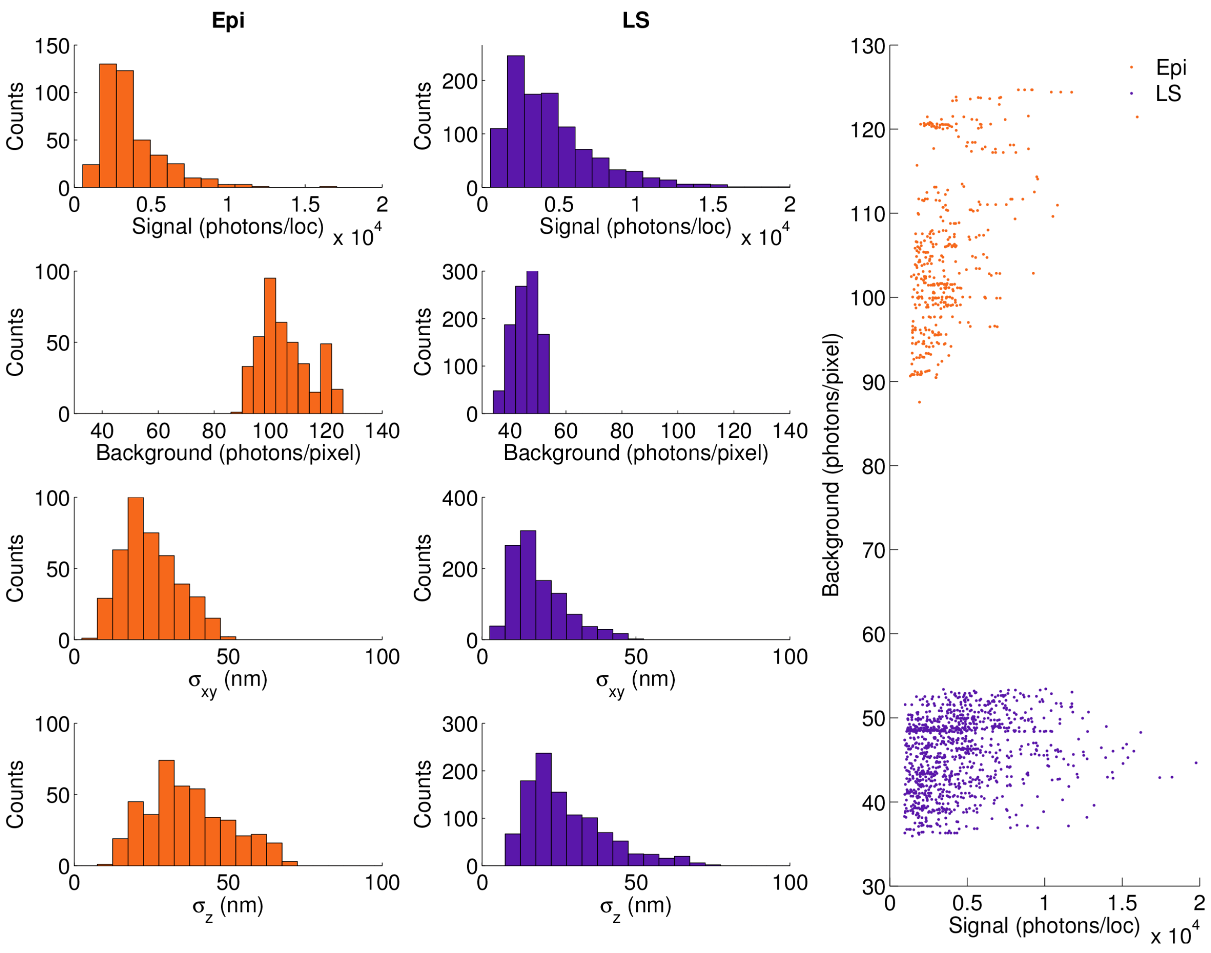

Supplementary Figure 9 Comparison between epi-illumination (Epi) and light sheet (LS) illumination for acquisition of single-molecule data using the double-helix PSF**.** Histograms showing the signal photons per localization, background photons per pixel, xy and z localization precision, and a direct comparison between the signal and background of localizations in the two cases. Using Epi/LS resulted in 413/1,061 localizations with median photons per localization of 3,181/3,857, background photons per pixel of 103/46, and localization precision of 23/16 nm and 35/24 nm in xy and z, respectively. The sample imaged was mitochondria (TOM20) in HeLa cells immunolabeled with Alexa Fluor 647.

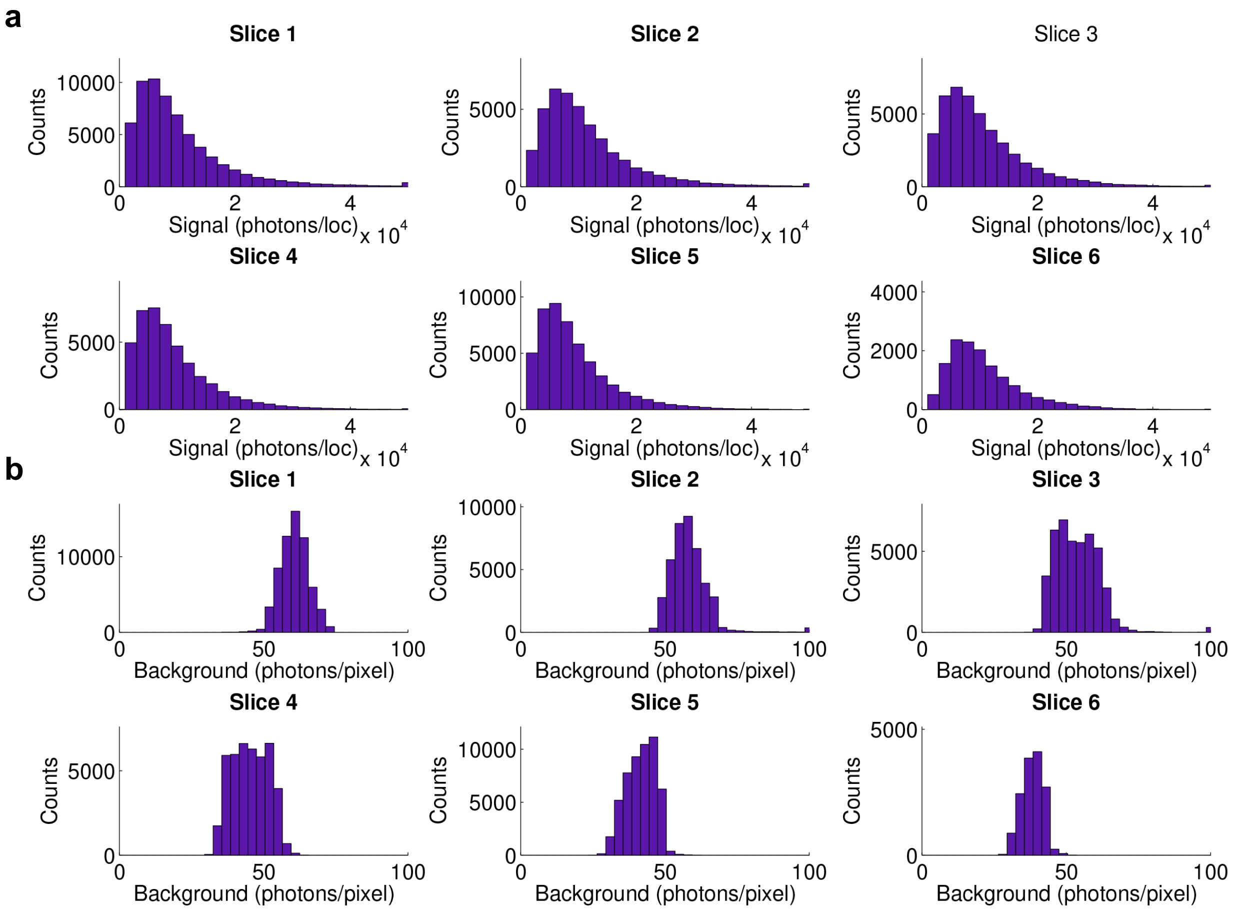

Supplementary Figure 10 Signal and background statistics of double-helix PSF localization data used for the 3D lamin B1 reconstruction**.** Histograms showing **a** signal photons per localization and **b** background photons per pixel for six different z-slices used in the reconstruction in **Figure 4c.** The median z position of the localizations in each slice was 1.1 µm, 1.7 µm, 2.9 µm, 3.7 µm, 4.7 µm, and 4.7 µm for slices 1-6, respectively. The data was filtered to remove localizations with a distance between lobes smaller than 5.5 pixels and larger than 10 pixels, and background photons per localization higher than 200. This resulted in ~525,000 localizations with a median of 8,465 ± 791 photons per localization and 50 ± 9 background photons per pixel. The values are given as the mean ± standard deviation of the median values in each z-slice.

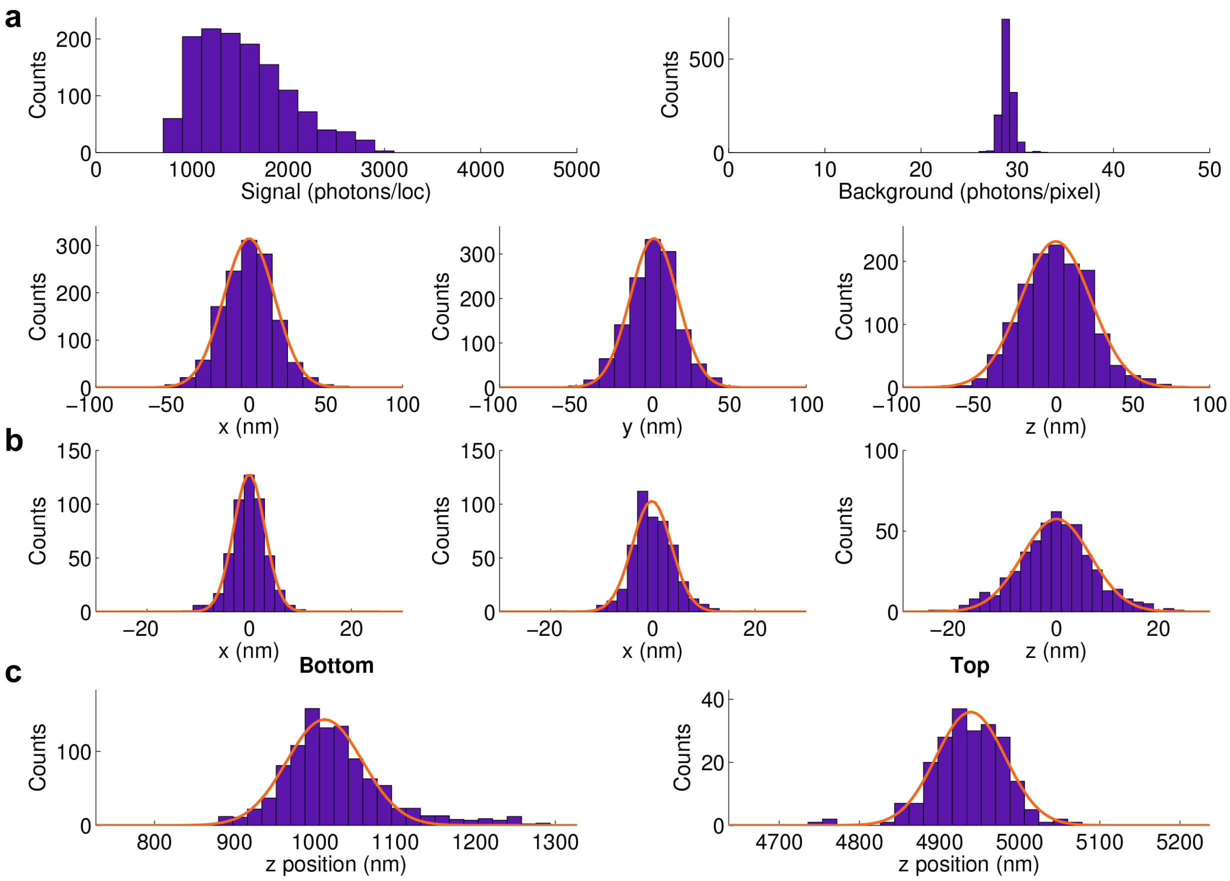

Supplementary Figure 11 Performance of long-axial range PSFs in thick cells**. a** Histograms showing the signal photons per localization, background photons per pixel, and x, y, and z positions of localizations of an isolated, stationary bead situated 3.3 µm above the coverslip detected using the double-helix PSF. The bead yielded a median of 1,467 photons per localization and 29 background photons per pixel. The histograms of the x, y, and z localizations were fit to 1D Gaussians (solid orange lines) and the localization precision in x, y, and z was estimated from the standard deviations of the fits. This resulted in an estimated average localization precision of 16 nm in xy and 23 nm in z. **b** Histograms showing the x, y, and z positions of the same isolated, stationary bead as in **a** detected using a 6-µm Tetrapod PSF in the second channel (the fiducial bead). Low-pass filtered data of this trace was subtracted before analysis. The histograms of the x, y, and z localizations were fit to 1D Gaussians (solid orange lines) and the localization precision in x, y, and z was estimated from the standard deviations of the fits. This resulted in an estimated average localization precision of 3 nm in xy and 7 nm in z. **c** Estimation of measured lamina thickness at the bottom and top of the nucleus. The thickness of the lamina at the bottom and at the top of the nucleus was estimated by fitting the localizations in z of small xy-regions at the bottom and top of the nucleus, respectively, to 1D Gaussians and calculating the FWHM of the fits. This resulted in estimated thicknesses of 113 nm and 101 nm for the bottom and top, respectively.

Supplementary Methods

Light sheet characterization. The width (defined here as the radius at $1/e^2$ of the peak intensity), r, and thickness (defined similarly), ω_0, of the light sheet were determined from directly imaging the excitation beam propagating through a fluorescent solution (1:1000 Alexa Fluor 647 conjugated secondary antibodies (ab150067, Abcam) in water). The thickness was measured after rotating the cylindrical lens by 90°. The cross-sectional profiles of the light sheet were measured by drawing a line perpendicular to the axis of light propagation across the resulting images and fitting the pixel intensities with a 1D Gaussian function. The thickness and width of the light sheet were determined to be 2.1 µm and 18.7 µm ($1/e^2$), respectively (Supplementary Fig. 2a). These parameters can be compared to the theoretically expected values of the width, r,

$$r = r_0 \frac{f_1}{f_{\text{cyl}}} \frac{f_{\text{obj}}}{f_2}, \tag{1}$$

and the thickness, ω_0,

$$\omega_0 = \frac{\lambda}{\pi \tan^{-1}\left(\frac{R}{f_{\text{obj}}}\right)}, \tag{2}$$

where r_0 = 1.1 mm is the (measured) beam radius ($1/e^2$) before the cylindrical lens, and f_{cyl} = 300 mm, f_1 = 35 mm, f_2 = 125 mm, and f_{obj} = 20 mm are the focal lengths of the cylindrical lens, the first and second lens in the illumination 4*f* system, and the illumination objective lens, respectively, λ = 0.647 µm is the wavelength of the excitation laser, and

$$R = r_0 \frac{f_2}{f_1} \tag{3}$$

is the radius of the beam ($1/e^2$) at the back aperture of the illumination objective in the axis not being focused by the cylindrical lens. By inserting these values of the various parameters into the formulas, the theoretical width and thickness were calculated to be 21.5 µm and 1.0 µm ($1/e^2$) respectively. While the theoretical width agrees reasonably well with the measured width, the discrepancy between the theoretical (1.0 µm) and measured thicknesses (2.1 µm) may be caused by scattering within the sample chamber or by imperfections of the illumination objective lens. In addition, in the current alignment of the optical setup, the back aperture of the objective is almost fully filled, i.e. R is not much smaller than the back aperture radius of the illumination objective lens. This means that the assumption used in equation 3 that the half cone angle of the light, θ, can be expressed as $\tan\theta = R/f_{\text{obj}}$ might not be completely accurate.

By using Gaussian beams, there is an inherent tradeoff between the depth of focus, or the confocal parameter, b, and the thickness of the light sheet. This is evident from the theoretical relation between b and ω_0:

$$b = \frac{2\pi\omega_0^2}{\lambda}, \tag{4}$$

which shows that b scales with ω_0 squared. Using extremely thin light sheets thus limits the useful field of view that can be imaged. To estimate the confocal parameter of our light sheet, we decreased the tilt of the beam and imaged the beam profile using an imaging objective with 20x magnification (1-UB525, 20x, NA 0.5, Olympus). We then used the Gaussian beam propagation equation to fit the thickness of the light

sheet, ω, as a function of the position x along the direction of beam propagation as measured from the focus,

$$\omega(x) = \omega_0 \sqrt{1 + \left(\frac{x}{z_{\mathrm{R}}} \right)^2} . \tag{5}$$

Here z_{R} is the Rayleigh length, which relates to the confocal parameter as $b = 2z_{\mathrm{R}}$. Fitting the data using this equation allowed us to experimentally determine z_{R} = 36.7 µm and ω_0 = 2.5 µm for the light sheet (Supplementary Fig. 2b). Inserting ω_0 = 2.5 µm into equation 5 results in a theoretical confocal parameter of 60.7 µm, corresponding to a theoretical Rayleigh length of 30.4 µm. This is in reasonable agreement with our data. The corresponding theoretical confocal parameter for a beam with ω_0 = 2.1 µm is 42.8 µm. These calculations show that our light sheet beam can be described using the Gaussian beam propagation equation, and that the light sheet has a usable range of more than 40 µm in the direction of propagation of the light sheet. This allows for measurements of most features of interest in mammalian cells without scanning of the sample. Measuring the y width of the light sheet across a range of 340 µm in the direction of beam propagation resulted in an experimental value of 19.1±0.1 µm, demonstrating that the width remains relatively constant across the entire field of view. All fits were performed with a non-linear least square algorithm in MATLAB.

The light sheet was tilted by positioning the illumination objective and the lens and mirror just before it such that the light path was inclined by about 10° with respect to the vertical (Supplementary Fig. 1). The resulting tilt of 10° with respect to the horizontal in the sample chamber was measured from the side of the chamber when propagating the light sheet beam through a fluorescent solution (1:1000 Alexa Fluor 647 conjugated secondary antibodies (ab150067, Abcam) in water). This tilt allowed the light sheet to be introduced into the sample chamber far above the aberrating interface of the bottom coverslip. In addition, the light sheet could still be focused all the way to the coverslip which is useful for imaging the entirety of adherent cells. The tilt will cause the light sheet to illuminate one side of the cell about 2-3 µm higher up than the other side. However, this is not a problem when combining the light sheet illumination with long axial range PSFs, since any fluorescent molecule within the entire illuminated range will be detected. It is important to highlight that the absolute axial position of each single-molecule emitter is encoded in the shape of the PSF rather than in the position (or thickness) of the light sheet.

Translation of light sheet using a motorized mirror. Translation of the light sheet in the lateral and axial direction in the sample chamber was achieved by tilting a 2D motorized mirror (8821 mirror mount with 8742 Picomotor controller, Newport) positioned in a conjugate plane to the back aperture of the illumination objective. The specifications of the motorized mirror for the maximum and minimum tilt angles are θ_{max} = 5° and $\theta_{\mathrm{min}} = 4{\cdot}10^{-5}$ °, respectively. The theoretical maximum and minimum displacements, Δ, in the sample plane can be calculated according to following equation:

$$\Delta = f_1 \tan\theta \frac{f_{\mathrm{obj}}}{f_2} . \tag{6}$$

By inserting the setup parameters into equation 7, the maximum and minimum displacements are calculated to be Δ_{max}= 490 µm and Δ_{min}= 3.9 nm. These calculations were experimentally validated by imaging the light sheet propagating through a fluorescent solution (1:1000 Alexa Fluor 647 conjugated secondary antibodies (ab150067, Abcam) in water) while moving the motorized mirror in steps each spanning

1000 θ_{min}. At each position, the fluorescence caused by the light sheet was imaged for 45 frames (50 ms per frame). The center and width of the light sheet were determined by fitting the cross-sectional profile in each frame with a 1D Gaussian function and calculating the average of those 45 positions. The measured displacement was calculated as the difference between the mean positions of the center of the light sheet at each step. These measurements resulted in a displacement, $\Delta_{meas} = 4.45 \pm 0.11$ µm per step (consisting of 1000 θ_{min}). This is in good agreement with the theoretical value of 3.9 µm per 1000 θ_{min} step (Fig. 3 and Supplementary Fig. 2c).

The total time required to complete a full step (1000 θ_{min}) was measured to 0.56 ± 0.02 s. During a total scan of 45 µm, the y width of the light sheet was measured to 17.8 ± 0.6 µm ($1/e^2$), showing that the width remained relatively constant across the scanned field of view.

Engineered point spread functions for 3D single-molecule localization. In this work, we have used the DH-PSF and the Tetrapod PSFs. The DH-PSF consists of two lobes instead of just one, where the midpoint between the two lobes reports on the xy position and the z position is determined from the angular orientation of the line connecting the center of the lobes. The phase pattern encoding for the DH-PSF varies rapidly across the pupil plane and was implemented using a transmissive dielectric phase mask (Double-Helix Optics, LLC). The recently developed Tetrapod PSFs contain more complex patterns, roughly tracing out the shape of a tetrapod when envisioned in 3D (Supplementary Figs. 4 and 5). They have been designed and optimized to be maximally informative over a specified signal, background, and axial range using Fisher information to find the best superposition of Zernike polynomials to form their phase pattern. A comparison between the performance (best-case localization precision as determined by Cramér-Rao lower bound) of various 3D PSFs, including the DH-PSF, the Tetrapod PSFs, and the astigmatic PSF, can be found in Refs.[1, 2]. The maximized information content of the Tetrapod PSFs in combination with the tunability of their axial range and design wavelength makes them extremely useful for imaging of sparse, bright emitters in thick samples[2]. This is the reason why we used these PSFs for fiducial imaging. Since the Tetrapod phase patterns are smoothly varying, they can be implemented using a DM, as well as a transmissive mask or a liquid crystal spatial light modulator. In this work, a Tetrapod PSF with 10 µm axial range and a designed wavelength of 660 nm was implemented using a DM and a Tetrapod PSF with 6 µm axial range and a designed wavelength of 550 nm was implemented using a transmissive phase mask for imaging of fiducial beads.

Fabrication of transmissive Tetrapod phase mask. Transmissive dielectric 6-µm Tetrapod phase masks were fabricated using standard photolithography methods at the Stanford Nanofabrication Facility. The phase mask fabrication can also be outsourced to a company.

Amorphous quartz wafers (4-inch diameter, ~475 µm in thickness, WRS Materials) were cleaned in a chemical bath of Piranha solution (90% sulfuric acid, 10% hydrogen peroxide) at 70°C for 20 min. The wafers were then rinsed with clean water in an automatic dump rinser and a spin/rinse dryer. Next, the wafers were placed in a Yield Engineering Systems (YES) oven to be dehydrated at 150°C before being primed with hexamethyldisilazane (HDMS) at 70°C for 20 min. After being primed, the wafers were spin-coated with a 1.6-µm thick layer of Shipley 3612 positive photoresist with a 5-mm edge bead removal by using a standard recipe on a Silicon Valley Group (SVG) resist coat system. Any excess solvent was driven off by baking the wafers on a hot plate at 90°C for 120 seconds. Patterning of the photoresist film was done with a KarlSuss MA-6 aligner and the photoresist film was exposed to ultraviolet (UV) illumination for 1.6 seconds. The patterns on the binary masks used in this exposure step were created by converting the

designed Tetrapod phase mask into three different binary masks (3 bits = 8 levels) with phase mask diameters of 2.7 mm. A vendor was used to print these binary mask patterns. To set the photoresist after the UV exposure, the wafers were baked on a hotplate at 110°C for 90 seconds. The positive photoresist in the UV-exposed regions was then removed by using a standard procedure on the SVG developer. Next, to drive off moisture and harden the remaining photoresist, the wafers were placed in a 90°C oven for 20 min. The etching of the quartz wafers was carried out with an Applied Materials Precision 5000 Etcher with the magnetically-enhanced reactive ion etch (MERIE) system. The duration of each etching period was calibrated and adjusted for each of the three etch steps. After the etch step, any photoresist left on the wafers was stripped with a Gasonics aura plasma asher. To be sure that any remaining photoresist was removed, the wafers were placed in a chemical bath of PRS-3000 at 60°C for 20 min. The etched and cleaned wafers were then rinsed with clean water in an automatic dump rinser and a spin/rinse dryer. After stripping any remaining photoresist, the 3D surface profiles of the phase masks on the quartz wafers were characterized with a Sensofar S Neox 3D optical profiler. This tool uses white-light vertical scanning interferometry to measure the step heights. After measuring the step heights, this entire conventional lithography process is repeated, starting with priming the clean and etched quartz wafers with HDMS again until all three etch steps were completed with the three different binary masks. Finally, after all three etch steps had been completed and measured, the Tetrapod phase masks were cut out of the 4-inch quartz wafer with a DISCO wafer saw.

Analysis of 2D data. 2D single-molecule localization was performed using the ThunderSTORM[3] plugin in FIJI[4] using wavelet filtering for background subtraction and a weighted least-squares fitting routine. The analysis methodology for extracting the 3D position of the fiducial bead imaged with a 10-µm Tetrapod PSF is described in section **Analysis of Tetrapod data**. The resulting data was filtered to remove localizations with a number of photons per localization higher than 10,000 and localization precision larger than 35 nm. This resulted in ~600,000 localizations with a median of 2,530 photons per localization, 28 background photons per pixel, and a 2D localization precision of 19 nm (Supplementary Fig. 6b). The localizations were reconstructed as 2D histograms in MATLAB using 16x16 nm^2 bins (Supplementary Fig. 6a). Gaussian fitting of a line scan over the localizations resulted in a measured nuclear lamina width of 130 nm (FWHM).

Analysis of Tetrapod data. Calibration and fitting analysis of Tetrapod images for drift correction was performed using a modified version of the open-source Easy-Pupil-Finder software[5] (https://sourceforge.net/projects/easy-pupil-finder/). Since the fiducial beads used in our experiments can be considered to be isotropic emitters, localization analysis of Tetrapod PSF data was performed using an imaging model based on the Gibson-Lanni scalar diffraction approximation for mismatched refractive indices[66-9]. In the model used here, the PSF was calculated from the pupil plane electric field. For an on-axis point source located at the coverslip surface and coincident with the focal plane of the objective, this field is given by

$$P(\rho,\varphi) = \frac{\operatorname{circ}(\rho)}{\left[1-\left(\frac{NA}{n_1}\rho\right)^2\right]^{1/4}} \exp\left[i\mathcal{M}(\rho,\varphi)\right] \tag{7}$$

where $\rho \in (0,1]$ is the normalized radial coordinate of the pupil plane, φ is the azimuthal coordinate, NA is the numerical aperture, n_1 is the refractive index of the immersion medium, and $\mathcal{M}(\rho,\varphi)$ is the particular Tetrapod phase pattern. The circle function limits the field to spatial frequencies in the passband of the objective, while the denominator accounts for compression of the spherical wavefront onto the pupil plane[10].

In a sample with a planar index mismatch created by the boundary between the coverslip/immersion medium (n_1=1.518) and sample ($n_2 \approx 1.33$), the axial position of an emitter is determined by two parameters: (1) its distance from the coverslip, z_{em}, and (2) the distance of the nominal focal plane of the objective from the coverslip, f_{nom}, corresponding to physical translation of the objective from perfect focus at the coverslip surface. Each parameter is associated with a corresponding phase term based on the accumulated phase lag of a ray of each spatial frequency in the pupil plane. The overall phase contributed by the two parameters is given by

$$\Psi_{depth}\left(\rho,\varphi \mid z_{\text{em}}, f_{\text{nom}}\right) = \frac{2\pi}{\lambda}\left[n_2 z_{\text{em}}\sqrt{1-\left(\frac{NA}{n_2}\rho\right)^2} - n_1 f_{\text{nom}}\sqrt{1-\left(\frac{NA}{n_1}\rho\right)^2}\right]. \tag{8}$$

Although emitters can be excited at different positions z_{em} in different lateral (x-y) regions throughout the field of view, parameterization of the depth-dependent phase in terms of the two distances z_{em} and f_{nom} describes the refractive index mismatch-induced aberrations for each unique point within the 3D sample volume. Inclusion of this depth factor produced qualitatively similar PSFs to those observed in experiment. However, the presence of slight optical aberrations in the setup led to reduced precision and accuracy in some regions of the axial range due to disagreement between the model and experimental PSFs[5]. In order to account for these aberrations, a phase retrieval algorithm[5] was used to determine a single additional phase factor, $\Psi_{\text{aberr}}(\rho,\varphi)$ appropriate for the entire *z*-range, which yielded a final pupil plane electric field given by

$$P_{\text{aberr}}(\rho,\varphi) = P(\rho,\varphi)\exp\left\{i\left[\Psi_{\text{depth}}\left(\rho,\varphi \mid z_{\text{em}}, f_{\text{nom}}\right) + \Psi_{\text{aberr}}(\rho,\varphi)\right]\right\}. \tag{9}$$

Phase retrieval was performed using calibration images of fiducial beads present in the samples, and separate calculations of Ψ_{aberr} were made for each sample and each PSF. A final but important consideration was the calibration of slight localization biases in both the lateral and axial directions, which arise due to the low symmetry of certain aberrations[11] and small outstanding differences between the model and experimental PSFs. These biases were calibrated by localizing images from a scan of the fiducial bead over the axial range of the Tetrapod PSF, in which the lateral position remained constant and the axial position was known. The bias, or offset between the estimated and known positions, was calculated in each dimension as a function of the axial position of the objective. Each localization was then corrected for biases in 3D using a polynomial interpolation of the bias evaluated at the estimated axial position.

Imaging and analysis of the Tetrapod PSFs for drift correction was performed in a few steps, as outlined in Supplementary Figure 7 and below. All localization using the phase-retrieved PSF models was done using a maximum-likelihood estimation routine assuming a Poisson noise model. (i) A phase-retrieved model was first obtained from a fiducial at the coverslip ($z_{\text{em}} = 0$). This model was used to localize fiducials within the sample volume and determine their height above the coverslip, z_{em}, by fixing the known value

of f_{nom} based on defocus of the objective lens. (ii) The calculated value of z_{em} was then fixed for each fiducial and the axial drift (via f_{nom}) was calculated and corrected every 10 s. (iii) After the experiment, a calibration scan was performed on the used fiducial bead. (iv) A new phase-retrieved model was calculated for the used fiducial bead and, in each relevant frame of experimental data, the fiducial was localized to retrieve the axial drift (via f_{nom}) as well as the lateral (x-y) drift over time. Drift data was low-pass filtered and interpolated in each dimension to produce final 3D drift data sets used for (v) subtraction from raw single-molecule localizations.

To estimate the localization precision of the axially highest fiducial fluorescent bead used for reconstructing the 3D lamin image using the 6-µm Tetrapod PSF, the x, y, and f_{nom} positions were low-pass filtered and the resulting low-pass filtered traces were subtracted from the x, y, and z localizations before analysis. The histograms of the resulting x, y, and z localizations were fit with 1D Gaussians and the localization precision in x, y, and z was estimated from the standard deviations of the fits. This resulted in an estimated average localization precision of 3 nm in xy and 7 nm in z (Supplementary Fig. 11b).

Analysis of double-helix data. Calibration and fitting analysis of DH-PSF images was performed using a modified version of the open-source Easy-DHPSF software[12] (https://sourceforge.net/projects/easy-dhpsf/). When analyzing images of the DH-PSF, the lobes of each PSF were fitted using non-linear least-squares functions in MATLAB with a pair of identical, radially symmetric 2D Gaussians as the objective function. The localization precision was calculated using the calibration described in Ref.[13]. The agreement of this calibration with the achievable localization precision in our new imaging platform was verified by imaging and localizing isolated, stationary molecules on a coverslip for typical signal and background photon numbers.

The data used for reconstructing the mitochondria images was filtered to remove localizations with a number of photons per localization higher than 20,000, distance between lobes smaller than 6 pixels and larger than 7.5 pixels, and z localization precision larger than 50 nm. This resulted in ~240,000 localizations with a median of 4,857 photons per localization, 46 background photons per pixel, and localization precisions of 13 nm and 20 nm in xy and z respectively (Supplementary Fig. 8b). The localizations were rendered using the Vutara SRX software from Bruker (Fig. 4a, b, Supplementary Fig. 8a, and Supplementary Movies 3-5).

Single-molecule data acquired with the DH-PSF comparing epi-illumination (Epi) and light sheet (LS) illumination were analyzed in the same manner as the data used for the 3D SR mitochondria reconstruction. The data was filtered to remove localizations with distances between lobes smaller than 6 pixels and larger than 7.5 pixels. Using Epi/LS resulted in 413/1,061 localizations with a median of 3,181/3,857 photons per localization, 103/46 background photons per pixel, and localization precision of 23/16 nm and 35/24 nm in xy and z, respectively, demonstrating the drastic improvement in localization precision when using light sheet illumination (Supplementary Fig. 9 and Supplementary Movie 6).

The data used for reconstructing the 3D lamin B1 image was filtered to remove localizations with a distance between lobes smaller than 5.5 pixels and larger than 10 pixels, and background photons per pixel larger than 200. This resulted in ~525,000 localizations with a median of 8,465 ± 791 photons per localization and 50 ± 9 background photons per pixel (Supplementary Fig. 10). The values are given as mean ± standard deviation of the median values in each z-slice. The median z position of the localizations in each slice was 1.1 µm, 1.7 µm, 2.9 µm, 3.7 µm, 4.7 µm, and 4.7 µm for slices 1-6, respectively. The localizations were rendered using the Vutara SRX software from Bruker, where spurious localizations were removed by means

of filtering for large average distance to eight nearest neighbors (Fig. 4c and Supplementary Movies 8-10). The localization precision 3.3 µm above the coverslip was estimated from repetitive localization of an isolated, stationary, green fiducial bead with some spectral bleed-through into the single-molecule far red channel. This dim bead yielded a median of 1,467 photons per localization and 29 background photons per pixel (Supplementary Fig. 11a). Using the equations for localization precision of the DH-PSF derived by Rieger *et al.*[14] and inserting typical PSF parameters and the median signal and background values of the bead and SM localizations, it was found that the localization precision of the bead should be worse than that for a typical single molecule. An estimation using the localizations from the bead should thus result in a conservative measure of the localization precision. Histograms of the bead localizations in x, y, and z were fitted with 1D Gaussians and the localization precision was estimated from the standard deviations of the fits. This resulted in an estimated average localization precision of 16 nm in xy and 23 nm in z. The thickness of the lamina at the bottom and at the top of the nucleus was estimated by fitting the localizations in z of small xy-regions at the bottom and top of the nucleus, respectively, to 1D Gaussians and calculating the FWHM of the fits. This resulted in estimated thicknesses of 113 nm and 101 nm for the bottom and top, respectively (Supplementary Fig. 11c).

Supplementary References

1. Shechtman, Y., Sahl, S. J., Backer, A. S. & Moerner, W. E. Optimal Point Spread Function Design for 3D Imaging. *Phys. Rev. Lett.* **113**, 133902 (2014).
2. Shechtman, Y., Weiss, L. E., Backer, A. S., Sahl, S. J. & Moerner, W. E. Precise 3D scan-free multiple-particle tracking over large axial ranges with Tetrapod point spread functions. *Nano Lett.* **15**, 4194-4199 (2015).
3. Ovesny, M., Krizek, P., Borkovec, J., Svindrych, Z. & Hagen, G. M. ThunderSTORM: a comprehensive ImageJ plug-in for PALM and STORM data analysis and super-resolution imaging. *Bioinformatics* **30**, 2389-2390 (2014).
4. Schindelin, J. *et al*. Fiji: an open-source platform for biological-image analysis. *Nat. Methods* **9**, 676-682 (2012).
5. Petrov, P. N., Shechtman, Y. & Moerner, W. E. Measurement-based estimation of global pupil functions in 3D localization microscopy. *Opt. Express* **25**, 7945-7959 (2017).
6. Backer, A. S., Backlund, M. P., von Diezmann, A. R., Sahl, S. J. & Moerner, W. E. A bisected pupil for studying single-molecule orientational dynamics and its application to 3D super-resolution microscopy. *Appl. Phys. Lett.* **104**, 193701-193705 (2014).
7. Gibson, S. F. & Lanni, F. Experimental test of an analytical model of aberration in an oil-immersion objective lens used in three-dimensional light microscopy. *J. Opt. Soc. Am. A* **8**, 1601-1613 (1991).
8. Kirshner, H., Aguet, F., Sage, D. & Unser, M. 3-D PSF fitting for fluorescence microscopy: implementation and localization application. *J. Microsc.* **249**, 13-25 (2013).
9. Lew, M. D., Backlund, M. P. & Moerner, W. E. Rotational mobility of single molecules affects localization accuracy in super-resolution fluorescence microscopy. *Nano Lett.* **13**, 3967-3972 (2013).
10. Richards, B. & Wolf, E. Electromagnetic Diffraction in optical systems. II. Structure of the image field in an aplanatic system. *Proc. R. Soc. London Ser. A* **253**, 358-379 (1959).
11. Carlini, L., Holden, S. J., Douglass, K. M. & Manley, S. Correction of a depth-dependent lateral distortion in 3D super-resolution imaging. *Plos One* **10**, e0142949 (2015).
12. Lew, M. D., von Diezmann, A. R. S. & Moerner, W. E. Easy-DHPSF open-source software for three-dimensional localization of single molecules with precision beyond the optical diffraction limit. *Protocol Exchange*, 026 (2013).
13. Gahlmann, A. *et al*. Quantitative multicolor subdiffraction imaging of bacterial protein ultrastructures in 3D. *Nano Lett.* **13**, 987-993 (2013).
14. Rieger, B. & Stallinga, S. The Lateral and Axial Localization Uncertainty in Super-Resolution Light Microscopy. *ChemPhysChem* **15**, 664-670 (2014).
