# Supplementary material for: 3D single-molecule super-resolution microscopy with a tilted light sheet
Source: Nat Commun. 2018 Jan 9;9:123. doi: 10.1038/s41467-017-02563-4 (PMC5760554; doi:10.1038/s41467-017-02563-4)
Supplement: Supplementary file 3 — Description of Additional Supplementary Files [file 41467_2017_2563_MOESM3_ESM.pdf]

Description of Additional Supplementary Files

File Name: Supplementary Movie 1
Description: Comparison between light sheet (LS) illumination and epi-illumination (Epi) for single-molecule imaging. The movie shows single molecules of Alexa Fluor 647, which were used to label the nuclear lamina protein lamin B1 in a HeLa cell. The fluorophores were imaged using a standard point spread function. Light sheet illumination clearly reduced the background as compared to epiillumination. The movie is shown at constant contrast and at live speed. Scale bar is 5 µm.

File Name: Supplementary Movie 2
Description: Single-molecule imaging using the double-helix point spread function. The movie shows single molecules of Alexa Fluor 647, which were used to label mitochondria in a HeLa cell. The double-helix point spread function has an axial range of ~2-3 µm and was implemented using a transmissive dielectric phase mask. The movie is shown at live speed. Scale bar is 3 µm.

File Name: Supplementary Movie 3
Description: 3D super-resolution reconstruction of mitochondria. Movie of reconstruction in Figure 4a (left) showing mitochondria (TOM20) in a HeLa cell immunolabeled with Alexa Fluor 647. Imaging of single molecules and fiducial beads was performed with the double-helix point spread function implemented using a transmissive dielectric phase mask.

File Name: Supplementary Movie 4
Description: Sectioning of mitochondria. Movie showing 100-nm thick z-slices of the 3D SR reconstruction in Figure 4a (left) showing mitochondria (TOM20) in a HeLa cell immunolabeled with Alexa Fluor 647. Imaging of single molecules and fiducial beads was performed with the double-helix point spread function implemented using a transmissive dielectric phase mask.

File Name: Supplementary Movie 5
Description: 3D super-resolution reconstruction of mitochondria. Movie of reconstruction in Figure 4a (right) showing mitochondria (TOM20) in a HeLa cell immunolabeled with Alexa Fluor 647. Imaging of single molecules and fiducial beads was performed with the double-helix point spread function implemented using a transmissive dielectric phase mask.

File Name: Supplementary Movie 6
Description: Comparison between light sheet (LS) illumination and epi-illumination (Epi) for 3D single-molecule imaging. The movie shows single molecules of Alexa Fluor 647, which were used to label mitochondria in a HeLa cell. The double-helix point spread function has an axial range of ~2- 3 µm and was implemented using a transmissive dielectric phase mask. Light sheet illumination clearly reduces the background as compared to epi-illumination. Statistics from single-molecule localizations from this data set are shown in Supplementary Figure 8. The movie is shown at live speed. Scale bar is 5 µm.

File Name: Supplementary Movie 7
Description: Comparison between single-molecule imaging using the double-helix point spread function at the bottom and top of the nucleus. The movie shows single molecules of Alexa Fluor 647, which were used to label lamin B1 in a HeLa cell. The double-helix point spread function has an axial range of ~2-3 µm and was implemented using a transmissive dielectric phase mask. The movie is shown at live speed. Scale bar is 5 µm.

File Name: Supplementary Movie 8
Description: 3D SR reconstruction of the entire nuclear lamina in a HeLa cell. Movie of reconstruction in Figure 4c (left) showing the lamina (lamin B1) in a HeLa cell immunolabeled with Alexa Fluor 647. Imaging of single molecules and fiducial beads was performed with the double-helix point spread function and a 6-µm Tetrapod point spread function, respectively, implemented using transmissive phase masks.

File Name: Supplementary Movie 9
Description: Sectioning of the entire nuclear lamina in a HeLa cell. Movie showing 100-nm thick z-slices of the 3D SR reconstruction in Figure 4c (left) of the entire nuclear lamina (lamin B1) in a HeLa cell immunolabeled with Alexa Fluor 647. Imaging of single molecules and fiducial beads was performed with the double-helix point spread function and a 6-µm Tetrapod point spread function, respectively, implemented using transmissive phase masks.

File Name: Supplementary Movie 10
Description: 3D SR reconstruction of lamin meshwork enveloping an intranuclear channel. Movie of reconstruction in Figure 4c (top right) showing a 1.3-µm thick y-slice of the 3D SR reconstruction of the nuclear lamina (lamin B1) in a HeLa cell immunolabeled with Alexa Fluor 647, where the lamin meshwork enveloping an intranuclear channel is visualized. Imaging of single molecules and fiducial beads was performed with the double-helix point spread function and a 6-µm Tetrapod point spread function, respectively, implemented using transmissive phase masks.
